# Association between Moral Distress and Burnout Syndrome in university-hospital nurses

**DOI:** 10.1590/1518-8345.6071.3747

**Published:** 2023-01-30

**Authors:** Camila Antunez Villagran, Graziele de Lima Dalmolin, Edison Luiz Devos Barlem, Patrícia Bitencourt Toscani Greco, Taís Carpes Lanes, Rafaela Andolhe

**Affiliations:** 1 Universidade Federal de Santa Maria, Departamento de Enfermagem, Santa Maria, RS, Brazil.; 2 Universidade Federal do Rio Grande, Departamento de Enfermagem, Rio Grande, RS, Brazil.; 3 Universidade Regional Integrada do Alto Uruguai e das Missões, Departamento de Enfermagem, Santigo, RS, Brazil.

**Keywords:** Nursing, Psychological Distress, Psychological Burnout, Occupational Health, Hospitals, Nurses, Enfermagem, Sofrimento Psicológico, Esgotamento Psicológico, Saúde do Trabalhador, Hospitais, Enfermeiras e Enfermeiros, Enfermería, Estrés Psicológico, Extenuación Psicológica, Salud Laboral, Hospitales, Enfermeras y Enfermeros

## Abstract

**Objective:**

to analyze the association between moral distress and Burnout Syndrome among nurses in a university hospital.

**Method:**

descriptive, analytical study conducted with 269 nurses working in a university hospital located in Rio Grande do Sul, Brazil. Data were collected in person in 2019 by previously trained collectors. A sociodemographic and employment questionnaire, the Brazilian Scale of Moral Distress in Nurses and the Maslach Burnout Inventory were applied. Descriptive and analytical statistical analysis was used.

**Results:**

an association was identified between moral distress intensity and frequency and its dimensions with Burnout Syndrome and its dimensions. Nurses with low professional achievement and high emotional exhaustion showed a higher prevalence of moral distress.

**Conclusion:**

an association between moral distress and Burnout Syndrome, as well as between their dimensions, was evidenced. The results suggest the need to investigate urgent interventions to mitigate the situations and manifestations of moral distress and Burnout Syndrome by developing strategies for workers’ health.

## Resumo

**Objetivo:**

analisar a associação entre sofrimento moral e síndrome de *Burnout* em enfermeiros de hospital universitário.

**Método:**

estudo descritivo-analítico, realizado com 269 enfermeiros atuantes em um hospital universitário localizado no Rio Grande do Sul, Brasil. A coleta de dados ocorreu de maneira presencial no ano de 2019 por coletadores previamente capacitados. Aplicaram-se questionário sociodemográfico e laboral, Escala Brasileira de Distresse Moral em Enfermeiros e o Inventário Maslach de *Burnout*. Empregou-se análise estatística descritiva e analítica.

**Resultados:**

identificou-se associação entre intensidade e frequência de sofrimento moral e suas dimensões com a síndrome de *Burnout* e suas dimensões. Enfermeiros em baixa realização profissional e alta exaustão emocional apresentaram prevalências mais elevadas para sofrimento moral.

**Conclusão:**

evidenciou-se a associação entre sofrimento moral e síndrome de *Burnout* bem como entre suas dimensões. Os resultados sinalizam a necessidade de investigar intervenções urgentes para amenizar as situações e as manifestações do sofrimento moral e a síndrome de *Burnout*, elaborando estratégias para a saúde dos trabalhadores.

## Resumen

**Objetivo:**

analizar la asociación entre sufrimiento moral y síndrome de *Burnout* en enfermeros de un hospital universitario.

**Método:**

estudio descriptivo y analítico, realizado en 269 enfermeros que actuaban en un hospital universitario localizado en el estado de Rio Grande del Sur, en Brasil. La recogida de datos se realizó de manera presencial en el año de 2019 por colectores previamente capacitados. Se aplicaron el cuestionario sociodemográfico y laboral, la Escala Brasileña de Estrés Moral en Enfermeros y el Inventario Maslach de *Burnout*. Se empleó el análisis estadístico descriptivo y analítico.

**Resultados:**

se identificó asociación entre intensidad y frecuencia de sufrimiento moral y sus dimensiones con el síndrome de *Burnout* y sus dimensiones. Los enfermeros que tuvieron baja realización profesional y alta extenuación emocional, presentaron prevalencias más elevadas para el sufrimiento moral.

**Conclusión:**

se evidenció asociación entre sufrimiento moral y síndrome de *Burnout* así como entre sus dimensiones. Los resultados señalan la necesidad urgente de investigar intervenciones para amenizar las situaciones y las manifestaciones del sufrimiento moral y el síndrome de *Burnout*, elaborando estrategias para la salud de los trabajadores.

**Highlights**

(1) There was a significant association between moral distress and the dimensions of Burnout syndrome.

(2) Moral distress intensity and frequency were are associated with Burnout Syndrome.

(3) Nurses showed a prevalence of moral distress.

(4) Moderate levels of moral distress intensity and frequency were found.

## Introduction

Nursing work is considered essential due to its performance in various health care settings. The nursing work process demands concentration and effort from workers, especially in hospitals, where a heavy workload is observed, which leads to illness^1^, such as physical and psychological fatigue, in addition to high stress levels^2^. 

These aspects may be related to situations in which nurses are faced with ethical issues that eventually cause conflicts and interfere in the quality of work activities, since they result in morally unsatisfactory responses^3^. Situations such as these can cause moral distress (MD), which occurs when a nurse feels unable to perform what he/she considers to be ethically correct, that is, he/she identifies what is appropriate to do, but realizes that he/she is unable to undertake such an action^4^. Furthermore, MD is defined as a psychological response to morally challenging situations, such as those of embarrassment and moral conflict^3^.

In Brazil, MD has been analyzed in different fields of nursing practice. In some hospital studies, low to medium levels of MD were found^5^ ^-^ ^6^. Recent studies have reported a moderate level of MD in hospital nurses^7^, which is mainly related to the work environment, such as the lack of resources and the lack of communication among professionals^8^. These aspects can threaten nurses’ health and well-being and lead them to illness, thus hindering their ability to provide safe, timely, efficient and people-centered care. In these cases, there may be implications for the professional, such as the development of Burnout Syndrome (BS).

BS is defined as a psychological syndrome characterized by three dimensions: emotional exhaustion, depersonalization and low professional achievement^9^. This topic has been investigated in health services, where health care professionals are exposed to high levels of stress at work, showing moderate to severe BS^10^. Another study reports that the workload may be related to the high risk for developing BS among nurses^11^.

The relationship between MD and BS has been pointed out in the literature, since MD development due to ethical problems, such as difficulty in making correct decisions and the lack of resources to provide proper care to patients causes stress, whose chronicity, in turn, leads to BS among nurses. Recent studies have reported a significant and positive relationship between MD and SB, showing that MD is consistently related to emotional exhaustion and depersonalization^3^ ^,^ ^12^. The main aspects of the work environment that are related to MD and BS are observed, such as: therapeutic obstinacy, disrespect for patients’ rights, nurses’ moral harassment, the work team’s incompetence, insufficient and/or inadequate communication, power relations, poor working conditions, work overload, (dis)satisfaction and intention to resign from one’s job^13^.

These situations, which are often found in daily work routine, raise concerns regarding the development of standards and resolutive strategies to promote these professionals’ health and quality of life. This study contributes to research and to nursing practice by identifying the association between two illness variables, which are relevant to the hospital setting and can interfere with nurses’ health. 

Given the above, the hypothesis is: Moral distress is associated with Burnout Syndrome among nurses at a university hospital. And, in order to verify it, the following objective was presented: to analyze the association between moral distress and Burnout Syndrome among nurses in a university hospital.

## Method

### Study design

This is a descriptive, analytical study guided by the STROBE (Strengthening the Reporting of Observational Studies in Epidemiology) tool.

### Research setting

The study was developed at a university hospital located in Rio Grande do Sul, Brazil. The institution provides 100% of its services through the Unified Health System and is characterized as a tertiary-level, general, public teaching hospital. The units that participated in this study were the: the adults’ and pediatric emergency department, internal medicine and surgery clinic, obstetric center and gynecological unit, intensive care units (adult, pediatric, neonatal and cardiology), outpatient clinic, hemato-oncology sector, pediatrics, psychiatry, surgery room, materials and sterilization center, recovery room, cardiovascular service and administrative sectors. 

### Population, selection criteria and sample definition

All the 303 nurses working in the hospital units were invited to participate in the study. The inclusion criterion adopted was: being a nurse who had been working at the hospital for at least one month. Nurses on leaves of absence for any reason during the data collection period were excluded. For analysis purposes, a minimum sample for a finite population was estimated, considering a population of 303 nurses, a 5% sampling error and an estimated percentage of 50%, which resulted in a minimum of 171 participants. A convenience sampling was defined in which all those who were working in their sectors were invited to participate. The final sample consisted of 269 nurses.

### Instruments used

The data collection instrument consisted of a sociodemographic and employment questionnaire, the Brazilian Scale of Moral Distress in Nurses (EDME-Br) and the Maslach Burnout Inventory (MBI). The sociodemographic and employment questionnaire was designed by this study’s authors, and contained the following items: gender; education; time elapsed since graduation; work shift; and employment relationship, namely under the Single Legal Regime (*Regime Jurídico Único* - RJU): for professional stability, and the Employment Contract System (*Consolidação das Leis do Trabalho* - CLT), which is characterized by the professional’s work relationship through a contract; leaves of absence from work; length of service for the institution; overtime; a receptive institution to dialogue; receptive management to dialogue; and the intention to resign from one’s job.

EDME-Br was originally developed in a Brazilian setting for hospital nurses, showing 0.98 reliability^14^. The scale has been validated for Brazil and authors were asked to authorize its use. The instrument consists of 49 items with a double six-point Likert scale to analyze MD intensity and frequency. The six constructs are: “Acknowledgement, power and professional identity” with 11 questions; “Safe and qualified care” with 11 questions; “Defense of values and rights” with 8 questions; “Working conditions” with 6 questions; “Ethical infractions” with 6 questions and “Work teams” with 7 questions. The instrument evaluates MD through the median and interquartile range in which (0 to 2) indicates low MD and (2.001 to 6) moderate to high MD^15^. 

MBI was developed in 1981^9^ and adapted for the Brazilian culture in 1995^16^, that is, the instrument has been validated for Brazil and the authors were asked to authorize its use for nurses in a hospital setting, showing a reliability level of 0.89. This instrument evaluates BS according to three dimensions: emotional exhaustion, depersonalization and low professional achievement. MBI features 22 questions divided into the scale’s three dimensions: Emotional exhaustion, consisting of nine questions (1, 2, 3, 6, 8, 13, 14, 16 and 20); Depersonalization, consisting of five questions (5, 10, 11, 15 and 22) and Low Professional Achievement, consisting of eight questions (4, 7, 9, 12, 17, 18, 19 and 21). 

The instrument consists of a five-point Likert scale with responses ranging from zero “never” to 4 “daily”, in which participants indicate how often they perceive or feel in relation to the statement in each question. The cutoff points for dividing the dimensions into high and low were obtained by the 75^th^ percentile for the emotional exhaustion and depersonalization dimensions and by the 25^th^ percentile for the professional achievement dimension, since it has the reverse score. 

### Data collection

Data were collected from April to June 2019 by previously trained collectors, all graduate students who were experienced in scientific research and data collection for quantitative studies. Nurses were approached in their work environment and they could choose to respond to the instrument on the spot or to hand it in at a later time, with prior scheduling for its collection. There were up to three attempts to collect the completed instrument on different days and at different times. When the instruments were being filled out, the collectors remained at a distance for the participants’ greater freedom. The collectors only approached the participants to collect the instruments and to resolve any doubts. The completed instruments were stored and protected in sealed envelopes, in order to maintain information confidentiality, and later delivered to the researcher in charge. The nurses who agreed to participate in the research were given information concerning the objectives and how to participate in the study, as well as the risks and benefits of the research.

They were also given the Informed Consent Form (ICF), which was signed by the participants and the researcher, with a copy for each one of those involved. This procedure ensured them the right to withdraw from participation at any time, without public disclosure of their information. 

### Data analyses

Data were organized by the Epinfo^®^ software, version 6.4, with two independent data entries and a check for errors and inconsistencies. For the analysis, the Statistical Package for the Social Sciences (SPSS), version 18.0 for Windows, was used. Descriptive statistics was utilized with absolute and relative frequency distribution for categorical variables and measures of central tendency and dispersion for quantitative variables. 

Normality distribution was analyzed by the Kolmogorov-Smirnov test. The Mann-Whitney test was performed to identify the association between the intensity and frequency of MD and its respective dimensions with burnout syndrome and its dimensions, being considered significant when P < 0.05. Subsequently, bivariate analysis was performed using the Chi-square or Fischer’s Exact test, in order to identify the sociodemographic and occupational variables and the burnout syndrome associated with moral distress, so as to select which of them would comprise the regression model. Thus, Poisson regression with robust and adjusted variance was applied and the prevalence ratios (PR) and their confidence intervals (95% CI) were estimated. Moral distress was considered to be the dependent variable. The following independent variables were included in the crude analyses: gender, overtime, employment relationship, education, leaves of absence from work, intention to resign from one’s job, work shift, receptive institution to dialogue, receptive management to dialogue, emotional exhaustion depersonalization and low professional achievement, with a P value <0.20. The variables showing a P value <0.15 were included in adjusted analysis 1 and, finally, those showing a P value <0.05 were included in adjusted analysis 2. A 5% significance level was adopted^17^ ^-^ ^18^.

As a form of analyzing the regression model’s quality, the multicollinearity indicators were examined by means of the linear regression procedure, tolerance measure and the variance inflation factor (VIF), through which no multicollinearity was registered, due to the fact that the tolerance and VIF values fell within levels higher than 0.1 and lower than 5, respectively, thus suggesting the model’s adequacy^18^.

### Ethical aspects

All ethical precepts of research involving human beings, as established in Resolution 466/12^19^, were observed. This study is part of a matrix project entitled “Moral distress in hospital nurses: what is its relationship with the ethical atmosphere and Burnout?”, which was submitted to the Local Research Ethics Committee for evaluation and approved according to report number 2.764.702.

## Results

The study participants were 269 nurses, of whom 88.1% (n=237) were females, 69.9% (n=188) had more than 10 years’ experience and 69.5% (n=187) had worked at the institution for longer than four years. 

Among the six factors in the EDME-Br scale, moderate levels of MD intensity and frequency were observed for items of three factors: “Working conditions” - item (10) “Acknowledging that the permanent equipment/materials available are insufficient” - frequency and intensity: (median 4.0/interquartile range: 2.0); “Safe and qualified care” - item (23) “Acknowledging insufficient access to the service by users” - frequency (median: 4.0/interquartile range: 2.0) and intensity (median: 4.0/interquartile range: 3.0) and “Work teams” - item (3) “Experiencing conditions of work overload” - frequency and intensity: (median 4.0/interquartile range: 2.0).

It is noteworthy that there was a prevalence of high emotional exhaustion and high depersonalization in 30.9% (n=83) and 24.9% (n=67), respectively and low professional achievement in 26.4% (n=71) of the nurses.

The association between MD intensity and BS is shown in Table 1. 

**Table 1:** Indices for the association of Moral Distress intensity and Burnout Syndrome in nurses (n=269). Rio Grande do Sul, Brazil, 2019

		F1 -API*			F2 -SQC^†^			F3 -DVR^‡^			F4 -WC^§^			F5 -EI^||^			F6 -WT^¶^			General MD**		
	N ^††^	Md ^‡‡^	IQI^§§^	U^||||^	Md^‡‡^	IQI^§§^	U^||||^	Md^‡‡^	IQI^§§^	U^||||^	Md^‡‡^	IQI^§§^	U^||||^	Md^‡‡^	IQI^§§^	U^||||^	Md^‡‡^	IQI^§§^	U^||||^	Md^‡‡^	IQI^§§^	U^||||^
EE^¶¶^	Low (186)	2.81	2.55	4823.0^||||^	3.36	2.11	6040.0	2.18	2.88	6263.0	3.83	2.00	5240.5^||||^	2.75	3.04	5723.0^||||^	3.14	1.86	4727.5^||||^	3.02	2.04	5171.0^||||^
	High (83)	4.00	1.91		4.00	1.91		3.00	2.63		4.66	1.67		3.66	2.67		4.14	1.86		3.84	1.74	
DP***	Low (202)	2.95	2.36	4708.0^||||^	3.36	2.09	5280.0	2.25	2.88	5171.0	3.83	2.00	5178.5^|^	2.66	2.88	5209.5	3.21	1.86	4669.5^||||^	3.07	2.01	4780.0^||||^
	High (67)	3.81	1.55		3.90	2.00		3.37	2.50		4.66	1.67		3.66	2.33		4.00	1.57		3.77	1.69	
PA^†††^	Low (71)	3.54	1.55	5136.0^||||^	3.90	1.91	5831.0	2.75	1.88	5946.5	4.33	1.67	5967.5	3.33	1.83	6216.0	3.71	1.71	5469.5	3.63	1.39	5654.0
	High (198)	3.00	2.36		3.36	2.20		2.31	3.03		3.83	2.17		3.00	3.54		3.28	1.89		3.13	2.20	
BS^‡‡‡^	Absent (250)	3.09	2.18	1144.5^||||^	3.40	2.18	1442.5^|^	2.37	2.88	1699.5	4.00	2.00	1673.5	3.00	2.86	1602.0	3.28	1.75	1257.5^||||^	3.20	1.95	1314.0^||||^
	Present (19)	4.36	1.27		4.36	1.36		3.37	2.88		4.66	1.50		4.00	2.00		4.57	1.57		4.13	1.27	

*F1 -API = Factor 1 - Acknowledgement, power, and professional identity; ^†^F2 -SQC = Factor 2 - Safe and qualified care; ^‡^F3 -DVR = Factor 3 - Defense of values and rights; ^§^F4 -WC = Factor 4 - Working conditions; ^||^F5 -EI = Factor 5 - Ethical Infractions; ^¶^F6 -WT = Factor 6 - Work teams; **MD = Moral distress; ^††^N = Number; ^‡‡^Md = Median; ^§§^IQI = Interquartile interval; ^||||^Mann Whitney U = Significance P < 0.01; ^¶¶^EE = Emotional exhaustion; ***DP = Depersonalization; ^†††^PA = Low professional achievement; ^‡‡‡^BS = Burnout Syndrome

There was an association between MD intensity and the emotional-exhaustion, depersonalization and low-professional-achievement dimensions, in which the nurses with high emotional exhaustion and high depersonalization showed higher MD intensity for both the general scale and the six factors. On the other hand, the nurses with low professional achievement and burnout showed higher MD intensity in Factor 1 - Acknowledgement, power and professional identity, in Factor 2 - Safe and qualified care, in Factor 6 - Work teams and in the general MD scale.

The association between MD frequency and BS is shown in Table 2. 

**Table 2:** Indices for the association of Moral Distress frequency and Burnout Syndrome in nurses (n=269). Rio Grande do Sul, Brazil, 2019

		F1-API*			F2 -SQC^†^			F3-DVR^‡^			F4 -WC^§^			F5 -EI^||^			F6 -WT^¶^			General MD**		
	N^††^	Md^‡‡^	IQI ^§§^	U^||||^	Md^‡‡^	IQI ^§§^	U^||||^	Md^‡‡^	IQI ^§§^	U^||||^	Md^‡‡^	IQI ^§§^	U^||||^	Md^‡‡^	IQI ^§§^	U^||||^	Md^‡‡^	IQI ^§§^	U^||||^	Md^‡‡^	IQI ^§§^	U^||||^
EE^¶¶^	Low (186)	2.09	2.00	4112.0^||||^	3.00	1.68	5700.0^||||^	1.37	1.63	5169.0^||||^	3.83	1.71	5246.0^||||^	2.00	2.00	5066.0^||||^	2.85	1.57	4647.0^||||^	2.56	1.44	4405.0^||||^
	High (83)	3.27	1.73		3.63	2.00		2.25	2.13		4.33	1.33		3.00	2.33		3.85	1.43		3.42	1.36	
DP***	Low (202)	2.18	1.91	4468.5^||||^	3.00	1.73	4635.0^||||^	1.37	1.66	4694.0^||||^	4.00	2.00	5464.0	2.08	1.88	4974.5^||||^	3.00	1.57	4570.5^||||^	2.65	1.43	4421.5^||||^
	High (67)	3.27	2.00		3.63	2.09		2.25	2.50		4.16	1.67		3.00	1.83		3.85	1.43		3.30	1.33	
PA^†††^	Low (71)	3.18	1.82	4709.5^||||^	3.72	1.82	4900.0^||||^	2.12	1.75	4953.0^||||^	4.16	1.50	5921.5	2.66	1.67	5767.0^|^	3.57	1.71	5133.0^||||^	3.23	1.06	4881.5^||||^
	High (198)	2.18	2.18		3.00	1.73		1.37	1.88		4.00	2.00		2.16	2.17		3.00	1.61		2.60	1.57	
BS^‡‡‡^	Absent (250)	2.31	2.02	905.5^||||^	3.04	1.82	1135.0^||||^	1.50	1.75	1270.5^||||^	4.00	1.88	1538.5	2.16	1.88	1351.5^|^	3.00	1.71	1098.5^||||^	2.73	1.41	978.0^||||^
	Present (19)	4.09	1.55		4.54	1.64		2.50	2.63		4.83	1.17		3.33	1.17		4.00	1.86		3.68	1.39	

*F1 -API = Factor 1 - Acknowledgement, power, and professional identity; ^†^F2 -SQC = Factor 2 - Safe and qualified care; ^‡^F3 -DVR = Factor 3 - Defense of values and rights; ^§^F4 -WC = Factor 4 - Working conditions; ^||^F5 -EI = Factor 5 - Ethical Infractions; ^¶^F6 -WT = Factor 6 - Work teams; **MD = Moral distress; ^††^N = Number; ^‡‡^Md = Median; ^§§^IQI = Interquartile interval; ^||||^Mann Whitney U = Significance P < 0.01; ^¶¶^EE = Emotional exhaustion; ***DP = Depersonalization; ^†††^PA = Low professional achievement; ^‡‡‡^BS = Burnout Syndrome

There was an association between MD frequency, the emotional-exhaustion and depersonalization dimensions and low professional accomplishment, in which nurses with high emotional exhaustion, high depersonalization and low professional achievement showed higher MD frequency for both the general scale and the six factors. 

In order to evaluate the associations found between MD, BS and the sociodemographic and employment variables, the prevalence and the raw and adjusted associations by Poisson Regression are shown in Table 3.

**Table 3:** Crude and adjusted regression analysis of Moral Distress, related to sociodemographic and employment variables and the Burnout Syndrome dimensions. (n=269). Rio Grande do Sul, Brazil, 2019

Variable	PRc^*^	CI^†^(95%)	p^‡^	PRad1^§^	CI^†^(95%)	p^‡^	PRad2^||^	CI^†^(95%)	p^‡^
*Sex*									
Female	1.065	0.970-1.171	0.118						
Male	1	-							
*Overtime*									
Yes	1.054	1.001-1.110	0.045	1.032	0.979-1.087	0.243			
No	1	-		1	-				
*Employment relationship*									
RJU^¶^	1.067	1.016-1.120	0.009	1.066	1.014-1.121	0.012	1.074	1.024-1.127	0.003
CLT^**^	1	-		1	-		1	-	
*Education*									
Nursing Degree	1.085	0.959- 1.228	0.195						
Graduate Certificate	0.992	0.874-1.125	0.895						
Master’s Degree	0.964	0.814-1.143	0.677						
Doctoral Degree	1	-							
*Leaves of absence from work*									
Yes	1.039	0.988- 1.092	0.13	1.017	0.970- 1.067	0.486			
No	1	-		1	-				
*Intention to resign from job*									
Yes	1.089	1.042-1.138	0.0001	1.054	1.005-1.104	0.029	1.064	1.017-1.114	0.008
No	1	-		1	-		1	-	
*Work shift*									
Morning	1.098	1.011-1.193	0.026	1.096	1.011-1.118	0.026	1.093	1.010-1.182	0.027
Afternoon	1.027	0.937-1.125	0.574						
Night	1.021	0.936-1.113	0.642						
Mixed	1	-		1	-		1	-	
*Receptive institution to dialogue*									
Yes	1.064	1.003-1.130	0.041	1.014	0.956-1.075	0.646			
No	1	-		1	-				
*Receptive management to dialogue*									
Yes	1.012	0.939-1.090	0.75						
No	1	-							
*Emotional exhaustion*									
Low	1								
High	1.110	1.064-1.158	1.082	1.035-1.131	<0.001		1.089	1.044-1.137	<0.001
*Depersonalization*									
Low	1.092	1.045-1.140	0.000	1.035	0.988-1.084	0.143			
High	1	-		1	-				
*Professional achievement*									
Low	1.119	1.076-1163	0.000	1.082	1.040-1.126	<0.001	1.098	1.057-1.141	<0.001
High	1	-		1	-		1	-	

* PRc = Crude prevalence ratio (P < 0.20); ^†^CI = Confidence interval (95%); ^‡^p = significance; ^§^PRad1= Adjusted prevalence ratio (P < 0.15) MD + overtime + leaves of absence + receptive to dialogue + depersonalization; ^||^PRad2 = MD + employment relationship + intention to resign + shift + emotional exhaustion + professional achievement (P < 0.05); ^¶^RJU = Single Legal Regime; **CLT = Employment Contract System

After the adjusted analysis, it was evident that nurses working in the morning shift, with an employment relationship under the Single Legal Regime (RJU) and who intended to resign from their jobs showed, respectively, a higher prevalence of 9%, 7% and 6% for MD when compared to the other variables. It was also observed that nurses with low professional achievement and high emotional exhaustion showed, respectively, a higher prevalence of 9% and 8% for MD.

## Discussion

This study showed an association between MD and BS as well as between their respective dimensions, finding that high emotional exhaustion, high depersonalization, and low professional achievement are related to decision-sharing among nurses and their colleagues, in addition to moral deliberation in difficult situations, which compromises the worker’s health^3^.

In this regard, considering the regression model, it was observed that the highest MD prevalence levels were among nurses on the morning shift, with an employment relationship under RJU, an intention to resign, high emotional exhaustion and low professional achievement. 

Nurses working the morning shift had a higher MD prevalence due to work overload resulting from the various routines occurring on this shift, such as baths, dressings, medical visits, in addition to internal interferences and clinical complications^20^. Professionals with an employment regime under RJU had a higher MD prevalence due to the lack of stability that such a contract poses on employees^21^. Corroborating these facts, it was observed in the literature that, despite the stability that the job provides, professionals under RJU contracts are more likely to resign from their jobs due to work overload and, consequently, to their experiencing more psychological distress and emotional exhaustion^22^. 

The intention to resign is justified by the higher MD prevalence and some factors may interfere, such as: long working hours, low wages, and difficulty in relating to other professionals. Thus, it is perceived that nurses who work more than 40 hours *per* week are more likely to leave their jobs when compared to those who work 40 hours *per* week or less^23^, which shows that each increase in MD frequency can double the chances of intending to resign^24^.

High emotional exhaustion among nursing professionals may be related to the severity of the patients’ conditions, workload increase and shortage of human resources^25^. These professionals’ work routine includes physical and emotional overload, human and material resources deficits, little recognition and lack of incentive for professional development^3^. 

Low professional achievement reflects a negative self-assessment of performance and motivation to act, showing a decreased feeling of competence and productivity, thereby adversely affecting the work environment^26^. Thus, these professionals felt unable to develop tasks, being less satisfied with their jobs due to the undervaluation of their work performance^27^.

The findings showed that “Working Conditions”, which refer to issues related to inadequate material resources and available equipment, are risks for the emergence of ethical conflicts, thus affecting care quality and, consequently, being an MD predictor. These findings express the occurrence of MD in work environments^5^ ^,^ ^15^.

The factor “Safe and qualified care” indicated problems regarding insufficient access to the service and educational actions, inadequate embracement and care provision impairment, unmet demands for care continuity and lack of resolutivity of health care actions. Professionals are prone to develop symptoms of emotional and physical exhaustion due to the work environment that involves complex demands, patient care expectations, and few resources available to provide proper care^28^.

The factor “Work teams” presented problems concerning the insufficient number of professionals to meet the demand, in addition to work overload and unprepared doctors. In agreement with these findings, a Brazilian study showed similar results, since this factor showed higher medians for MD intensity and frequency when compared to the others, as the insufficient number of professionals on a team causes work overload and, consequently, there cannot be proper patient care^15^.

This study showed an association between MD intensity (P < 0.001) and frequency (P < 0.001) and the BS dimensions. In intensive care units, nurses showed a medium-to-high degree of emotional distress and depersonalization when compared to those working in inpatient units^29^, which may be associated with the nurses’ exposure to intense working hours, feelings of demotivation to perform tasks and difficulties to relate with colleagues and patients, thus leading to higher MD intensity^30^. Regarding the relationship between the frequency of MD situations and the BS dimensions, it is shown that, from this perspective, studies suggest the need for resolute measures to mitigate illness among nurses^31^ as well as its interference with the quality of the care provided^32^.

As reported in the literature, regarding nurses working in hospitals and primary care in Punta Arenas, almost half of these health professionals had low or medium levels of professional achievement^33^. In a study, it was possible to observe that professional accomplishment negatively influences MD, showing that the higher the professional achievement, the lower the MD^34^. Low professional accomplishment at work can be described as a feeling that little has been achieved, causing a reduction in motivation and in the feeling of accomplishment^30^.

A limitation to this study is the fact that it was conducted in only one hospital, which renders it difficult to generalize its results.

However, it contributes to the advancement of knowledge in the nursing field by evaluating the association of MD frequency and intensity with the dimensions of BS. Its results suggest the need for developing actions that can reduce damage to workers’ health by addressing strategies for better institutional organization, personnel and material resources sizing in order to provide safe and qualified care. 

It is suggested that future publications should include studies involving other health care professionals in different settings and services, as well as, according to what was observed, intervention studies that will bring solutions to the institutions.

## Conclusion

Based on the analysis in this study, an association was found between MD and SB, as well as between their dimensions, in which MD intensity and frequency were associated with high emotional exhaustion, high depersonalization and low professional achievement. It was also observed that nurses working the morning shift, under the single legal regime, who intended to resign from their jobs and showed low professional achievement as well as high emotional exhaustion, had a higher MD prevalence.

The findings in the present study indicate the need to investigate urgent interventions in order to mitigate the situations leading to MD and BS and their manifestations by developing strategies for workers’ health. It is expected that the present study will contribute to understand and inform about the importance of MD and BS implications in workers’ health, as well as in patient care.
